# Analysis of genetic diversity and population structure in *Asparagus* species using SSR markers

**DOI:** 10.1186/s43141-020-00065-3

**Published:** 2020-09-14

**Authors:** Manish Kapoor, Pooja Mawal, Vikas Sharma, Raghbir Chand Gupta

**Affiliations:** 1 Department of Botany, Punjabi University Patiala, Patiala, Punjab 147002 India; 2 Department of Botany, Sant Baba Bhag Singh University, Khiala, Jalandhar, Punjab 144030 India

**Keywords:** *Asparagus*, Simple sequence repeat (SSR), Genetic diversity, Phylogenetic relationship

## Abstract

**Background:**

Various *Asparagus* species constitute the significant vegetable and medicinal genetic resource throughout the world. *Asparagus* species serve as important commodity of food and pharmaceutical industries in India. A diverse collection of *Asparagus* species from different localities of Northwest India was investigated for its genetic diversity using simple sequence repeat (SSR) markers.

**Results:**

Polymorphic SSR markers revealed high genetic diversity. Primer SSR-15 amplified maximum of 8 fragments while 3 primers, namely, SSR-43, SSR-63, and AGA1 amplified minimum of 3 fragments. Collectively, 122 alleles were amplified in a range between 3 and 8 with an average of 5 alleles per marker. The size of the amplified alleles ranged between 90 and 680 base pairs. Polymorphism information content (PIC) value varied from a highest value of 0.499 in primer AGA1 to a lowest value of 0.231 in primer SSR-63 with a mean value of 0.376 showing considerable SSR polymorphism. Dendrogram developed on the basis of Jaccard’s similarity coefficient and neighbor-joining tree segregated all the studied *Asparagus* species into two discrete groups. Structure analysis based on Bayesian clustering allocated different accessions to two independent clusters and exhibited low level of individual admixture.

**Conclusions:**

The genetic diversity analysis showed a conservative genetic background for maximum species of asparagus. Only Accessions of *Asparagus adscendens* were split into two diverse clusters suggesting a wide genetic base of this species as compared to other species. Overall genetic diversity was high, and this germplasm of Asparagus can be used in future improvement programs. The findings of current research on *Asparagus* germplasm can make a momentous contribution to initiatives of interbreeding, conservation, and improvement of *Asparagus* in future.

## Background

The genus *Asparagus* belongs to the recently created family *Asparagaceae* and reported to be comprised of about 300 species distributed all over the world. Of these, 22 species have been reported in India. *Asparagus* species are distributed throughout temperate, tropical, and subtropical parts of India [1]*.* Some of the *Asparagus* species distributed and cultivated in North India are *A. racemosus*, *A. adscendens*, *A. officinalis*, *A. plumosus*, *A. sprengeri*, *A. virgatus*, *A. filicinus*, *A. falcatus*, *A. pyramidalis*, *A. retrofractus*, etc. [2, 3]. The most recent intrageneric classification split *Asparagus* species into three subgenera: *Asparagus*, *Myrsiphyllum*, and *Protasparagus*. All dioecious species bearing unisexual flowers fall in the *Asparagus* subgenus while hermaphrodite species are included into the subgenera *Protasparagus* and *Myrsiphyllum*. *Asparagus* species grow as perennial herbs, delicate woody shrubs, and climbers. They are provided with short underground rhizomes from which the aerial shoots arise. They can be propagated by division of clump or rhizome and seeds. Roots are often tuberous, sometimes fleshy. Shoots vary from low herbs to stout woody vines reaching 15 m or more long. Leaves reduced to scale-like bracts, often spiny. Members of the genus are characterized by stem modifications called as cladodes, which are leaf-like organs. Flowers appear usually axillary or terminal in group of 1–4. Sometimes umbellate, often racemose on special branches lacking cladodes. Perianth is 6-parted. Fruit is berry. Due to dioecy in *Asparagus*, cross-pollination is obligate except for occasional self-pollination in perfect flowers occurring on andro-monoecious plants [4]. Wind is not a factor in pollination. Bees and primarily honeybees are pollinators [5, 6]. Cytological reports indicate that polyploidization is common in this genus and considered an important mechanism in the evolution of *Asparagus* [7–11]*.*

Though several species of the genus are grown as ornamentals in India*,* many of *Asparagus* species are used as food and medicines. Of these, *A. racemosus and A. adscendens* are the most commonly used species in indigenous medicines. The medicinal properties of *Asparagus* are attributed to its main steroidal bioactive compounds such as saponins, sarsasapogenins, polyphenols, and flavonoids (kaempferol, quercetin, and rutin [12]. Roots of *Asparagu*s are the main source of drug shatawar [13]. It is widely used in about 64 ayurvedic formulations, apart as galactogogue. *A. racemosus* is most commonly used in traditional medicines. *A. racemosus*, commonly known as Shatavari meaning “curer of a hundred diseases,” an amazing herb, is called as the “Queen of herbs” [3]. It is mainly known for its phytoestrogenic properties and is a rasayana or rejuvenating herb having beneficial restorative effects in women’s complaints. Its saponins are extensively used in hormone replacement therapy in place of synthetic estrogens [14, 15]. Several of the so-called phytoestrogens have been linked with cancer prevention [16]. The *Asparagus* species show a large number of diverse biological activities (mainly associated with steroidal saponins), e.g., aphrodisiac, antioxidant, an immuno-stimulant, anti-cancerous, anti-bacterial, anti-diabetic, anti-depressant, anti-inflammatory, anti-hepatotoxic, anti-tuberculosis, and anti-diarrheal [12, 13, 17]. *A. officinalis* is a highly prized vegetable and is mainly consumed for its edible shoots called spears. It has strong anti-cancerous effect also [18]. Despite being highly significant genus, it is not well studied as compared to other genera. Therefore, it is very important to study the diversity of existing germplasm of *Asparagus* in India. These types of studies can be useful in identification of promising accessions for further improvement of the crop.

SSR markers also known as Microsatellites are tandem repeats of 1-6 base pairs in the nucleotide sequences of DNA. These are the valuable tools for various purposes such as assessment of genetic variability and relationships, fingerprinting, marker-assisted selection, breeding, genetic linkage mapping, population genetics, and evolutionary studies because of their reproducibility, highly polymorphic nature, multi-allelic nature, co-dominant inheritance, relative abundance, and good genome coverage. Due to recent developments in sequencing technologies and bioinformatics analyses, large number of less costly SSRs are produced. Multiple uses and immense therapeutic value of *Asparagus* species have attracted global attention. Thus, increasing demand of *Asparagus* and habitat destruction results in serious reduction in native populations and has been recognized as vulnerable. Hence, evaluation of genetic diversity becomes essential for the identification of diverse germplasm and development of suitable conservation, management, and multiplication strategies for the existing germplasm.

## Methods

### Plant material

Forty-eight accessions of 10 *Asparagus* species were collected from diverse localities of Northwest India and maintained in the Botanic Garden and plant conservatory of Department of Botany, Punjabi University Patiala, India. All the 48 accessions belong to ten *Asparagus* species namely *A. adscendens*, *A. racemosus*, *A. virgatus*, *A. retrofractus*, *A. densiflorus*, *A. officinalis*, *A. plumosus*, *A. sprengeri*, *A. pyramidalis*, and *A. falcatus* (Table 1). Out of these 10 *Asparagus species*, *A. adscendens* Roxb., *A. falcatus* L., and *A. racemosus* are native to India but rest of the species have been introduced from other countries. The introduced species have been widely naturalized in India and cultivated mainly in the tropical and subtropical regions of India.

**Table 1:** List of genotypes with their locations

S. no.	Genotype	Location	Altitude (m)	Latitude, Longitude
1	*A. adscendens 1*	J&K, Udhampur	755 m	32.93° N, 75° E
2	*A. adscendens 2*	Uttarakhand, Uttarkashi	1158 m	30.73° N,78.45° E
3	*A. adscendens 3*	J&K, Jammu	305 m	32.71° N, 74.87° E
4	*A. adscendens 4*	J&K, Jammu	305 m	32.71° N, 74.87° E
5	*A. adscendens 5*	J&K, Udhampur	755 m	32.93° N, 75° E
6	*A. adscendens 6*	J&K, Udhampur	755 m	32.93° N, 75° E
7	*A. adscendens 7*	J&K, Kathua	387 m	32.38° N, 75.51° E
8	*A. adscendens 8*	H.P., Solan	1502 m	30.905° N, 77.09° E
9	*A. adscendens 9*	J&K, Udhampur	755 m	32.93° N, 75° E
10	*A. adscendens 10*	H.P., Mandi	769m	31.70° N, 76.93° E
11	*A. adscendens 11*	J&K, Kathua	387 m	32.38° N, 75.51° E
12	*A. adscendens 12*	H.P., Mandi	769 m	31.70° N, 76.93° E
13	*A. adscendens 13*	H.P., Mandi	769 m	31.70° N, 76.93° E
14	*A. adscendens 14*	J&K, Udhampur	755 m	32.93° N, 75° E
15	*A. adscendens 15*	H.P., Bilaspur	673 m	31.34° N, 76.68° E
16	*A. adscendens 16*	J&K, Udhampur	755 m	32.93° N, 75° E
17	*A. adscendens 17*	H.P., Solan	1502 m	30.905° N, 77.09° E
18	*A. racemosus 1*	H.P., Solan	1502 m	30.905° N, 77.09° E
19	*A. racemosus 2*	J&K, Jammu	305 m	32.71° N, 74.87° E
20	*A. racemosus 3*	Punjab, Bathinda	211 m	30.20° N 74.95° E
21	*A. racemosus 4*	Punjab, Patiala	244 m	30.36° N, 76.45° E
22	*A. racemosus 5*	H.P., Solan	1502 m	30.905° N, 77.09° E
23	*A. racemosus 6*	H.P., Solan	1502 m	30.905° N,77.09° E
24	*A. racemosus 7*	Punjab, Patiala	244 m	30.36° N, 76.45° E
25	*A. racemosus 8*	Uttarakhand, Dehradun	435 m	30.31° N, 78.02° E
26	*A. racemosus 9*	Haryana, Bhiwani	225 m	28.77° N, 75.99° E
27	*A. racemosus 10*	Punjab, Sangrur	235 m	30.36° N, 75.86° E
28	*A. racemosus 11*	Delhi	250 m	28.36° N, 77.13° E
29	*A. racemosus 12*	Rajasthan, Udaipur	600 m	24.58° N, 73.68° E
30	*A. racemosus 13*	Rajasthan, Jhunjhunu	323 m	28.13° N, 75.4° E
31	*A. virgatus 1*	J&K, Jammu	305 m	32.71° N, 74.87° E
32	*A. adscendens 18*	J&K, Kathua	387 m	32.38° N, 75.51° E
33	*A. retrofractus 1*	J&K, Jammu	305 m	32.71° N, 74.87° E
34	*A. retrofractus 2*	Punjab, Sangrur	235 m	30.36° N 75.86 °E
35	*A. officinalis 1*	H.P., Solan	1502 m	30.905° N,77.09° E
36	*A. officinalis 2*	J&K, Jammu	305 m	32.71° N, 74.87° E
37	*A. officinalis 3*	Delhi	250 m	28.36° N, 77.13° E
38	*A. densiflorus 1*	J&K, Jammu	305 m	32.71° N, 74.87° E
39	*A. densiflorus 2*	H.P., Solan	1502 m	30.905° N, 77.09° E
40	*A. densiflorus 3*	Punjab, Patiala	244 m	30.36° N, 76.45° E
41	*A. falcatus1*	Haryana, Bhiwani	225 m	28.77° N, 75.99° E
42	*A. falcatus2*	Punjab, Patiala	244 m	30.36° N, 76.45° E
43	*A. plumosus 1*	Punjab, Patiala	244 m	30.36° N, 76.45° E
44	*A. plumosus 2*	J&K, Jammu	305 m	32.71° N, 74.87° E
45	*A. sprengeri 1*	H.P., Solan	1502 m	30.905° N, 77.09° E
46	*A. sprengeri 2*	Haryana, Bhiwani	225 m	28.77° N, 75.99° E
47	*A. adscendens 19*	J&K, Kathua	387 m	32.38° N, 75.51° E
48	*A. pyramidalis 1*	Chandigarh	320 m	30.73° N, 76.47° E

Identification of the collected plants was done by taxonomist Prof. M. Sharma as per Bentham and Hooker [19] system by consulting different ‘Floras,’ such as ‘The Standard Cyclopedia of Horticulture’ vol.1 [20] and other Floras like ‘Flora of Patiala’ [21], ‘Flora of Himachal Pradesh vol. III’ [22], ‘Flora of Sirmaur District’ [23], and ‘Flora of Kullu District’ [24]. Further, the authentic confirmation of the collected specimens was done by comparing them with authentic specimens available at Botanical Survey of India (BSI), Dehra Dun, Forest Research Institute, Dehra Dun (FRI) and Herbaria of Punjabi University, Patiala (PUN). The voucher number for each species is given in supplementary Table 1.

### DNA extraction

DNA was extracted from 1 g of fresh young cladodes by the slightly modified CTAB method [25]. DNA stock solutions were prepared using T_10_E_1_ buffer. DNA was quantified by 0.8% agarose gel and compared to standard lambda DNA (Fermentas, Lithuania) and dilutions were made to maintain equal final concentration of each DNA sample, i.e., 13 ng/μl.

### Data mining and primer designing

Nucleotide sequence data of Asparagus species were downloaded from NCBI on February 2016 and checked for redundancy. The non-redundant sequences were assembled using EGassembler online software and the assembled sequence data was then utilized to search SSR motifs using The SSR contacting sequences were subjected to Primer3 software for designing primers from flanking sequences. Finally, 20 primers were synthesized and used for validation, and polymorphic 8 primers were used in this study with other sixteen primers developed by others in different Asparagus species [26, 35].

### SSR genotyping

Of 40, 15 SSR primers (PN1-PN15) were newly designed and rest were adopted from related *Asparagus* species (*A. officinalis*) [26, 35]. Out of 40, 24 polymorphic SSR primers were used to achieve PCR amplifications in Veriti™ 96-Well Thermal Cycler (Applied Biosystems, CA, USA), in a 12.5 μl reaction volume as per Sharma et al. 2009 [27]. The ingredients present in reaction mixture were 2 μl genomic DNA (13 ng/μl), 1.25 μl 10× PCR Buffer (10 mM Tris-HCl, 50 mM KCl, pH 8.3), 1.0 μl MgCl_2_(25 mM), 1.0 μl dNTP mix (0.2 mM each of dATP, dGTP, dTTP, dCTP ), 0.5 μl of each of two primers and 0.1 μl Taq DNA polymerase (5 U/μl). PCR reactions were carried out with the program: (1) 4 min of initial denaturation at 94 °C, (2) 35 cycles of run, each with denaturation at 94 °C for 1 min, annealing at 49–57 °C (depending on annealing temperature of different primer pairs (Table 2) for 1 min and extension at 72 °C for 1 min, (3) followed by a final step of extension at 72 °C for 7 min. All the PCR amplification products were first checked on 3% agarose gel, stained with ethidium bromide dye, and then run on 6% polyacrylamide gel in 1× TBE buffer, at a constant 65 W for 90 min at room temperature. SSR fragments were visualized using silver-staining. Alleles were sized by 50 bp DNA ladder (Fermentas, Lithuania).

**Table 2:** Characterized SSR primers with their diversity characteristics. PN series primers are newly developed primers in the present study

Primer ID	Primer sequence (5´–3´)	Repeat motif	T_**a**_ (°C)	No. of bands	Size range (bp)	PIC	Ho	He
SSR-13	F:CGACCAGAGAAGGAAGGAG R:CAACCACGCTCATAAGAAC	(TC)_11_	49	4	590–680	0.630	0.479	0.682
SSR-15	F:ATGATCCCTGAAGTTGTTG R:GTTCCTCTACCAGCCAAG	(TC)20(TG)_10_	47	8	90–200	0.628	0.646	0.668
SSR-22	F:TAAGCAACTCACTCACTATG R:TGATGTGTGAAGGAGGAGG	(CACT)_5_	48	7	350–470	0.790	0.708	0.820
SSR-37	F:TATGTTCCTTGCTTCCATG R:CGGTAGAAGTGATTGTGTAT	(AG)_11_	47	7	140–270	0.771	0.750	0.807
SSR-40	F:GCATATTTCTACTACGCCTCC R:CAAACTAACCCTCAATCACTCG	(ATAGA)_5_	52	5	400–440	0.576	0.167	0.622
SSR-43	F:CTTGATGGAGCTGGTCTTGT R:TTCTCCACCCTCAATCTCAATAC	(AG)_9_	52	3	250–400	0.630	0.458	0.696
SSR-56	F:GCTGCTAAGGGATATAGTGCCA R:TATGGTTGCAGAGGATAGGT	(GACAAT)_6_	50	5	180–350	0.760	0.542	0.800
SSR-63	F:TTAAGTCAGGTGGTGCTCTC R:CTGGATTAGTGGTTGATGATG	(TTGAAAA)_54_	50	3	140–200	0.585	0.083	0.658
SSR-69	F:GGCTAATTGTGTTGGGAATCG R:CCAACTAATCTACTGACACACG	(AG)_14_	52	5	220–400	0.699	0.583	0.750
SSR-77	F:GGCCTGCATGTTCTTTATATC R:GCTCATTCTCATCCACTCAT	(CA)_12_	50	6	230–300	0.679	0.333	0.726
SSR-83	F:GAGTTGAGGCGAGGGACAT R:GTTACTTTCGAGGAGGCCA	(AC)_10_	51	4	180–400	0.644	0.208	0.702
PN3	F: CCCCTCAAAATCTAACTTCTC R:CAGTTCTACATGCAGATGACC	(GGC)_7_	50	4	125–160	0.389	0.208	0.411
PN4	F:CCCCCTCTCTAGATATCGTC R:AAGAAGTCGAGGTTTCTGATG	(CT) _12_	50	6	500–620	0.743	0.667	0.785
PN9	F:TGAATCTATGGATACCGAAAA R:ATAAAGCCAGACACATCAACA	(TC) _12_	47	5	320–450	0.767	0.646	0.804
PN10	F:GATTTTTCTGCCTTCTCTACC R:ATTTTGCCCTTCCATTATCT	(CT)_8_	46	5	180–230	0.616	0.625	0.664
PN12	F:TGGCTTTTGTGTTGAAATACT R:ACAAATTTTCCCCAATTTTC	(GGT)_6_	44	5	280–450	0.641	0.729	0.702
PN13	F:TTTCTATTGCCGGAGACTAA R:GCAACTATTCTTCATCCACAG	(GAT)_6_	48	6	140–170	0.782	0.938	0.817
PN14	F:GACATCGATCTCCTTCTTCTT R:AATCCTGGCATCTGAGGT	(TC)_9_	48	5	400–600	0.763	0.333	0.802
PN15	F:CCGCTCAGAACTTGTTATTAT R:AATTACAAATAGCCTCTTTTGG	(TA)_16_	47	5	400–600	0.710	0.333	0.756
AGA1	F: CCGGTGCTTTGATTACTGCT R: GATCATCATCTTGCGCATTG	(AGA)_11_	50	3	480–600	0.536	0.479	0.598
AG5	F: GATTAATAAAGCGCCGCTGA R:ACATAAGCCCATACTTGCGG	(TC)_18_	50	5	490–640	0.784	0.396	0.820
TC1	F: AGGTGGAGAACAAATGGCTG R:CGAGCTCAATTGAAATCCATAA	(TC)_12_	49	5	150–250	0.789	0.479	0.824
TC3	F: CACCATTTCAAATCCCCACT R:GAGGCTAGAGCTCCGCTCAT	(AG)_13_	50	7	150–350	0.775	0.750	0.809
TC8	F: GGCTAGCCGAAAGAATCTCC R:TCTTCCTCCTCCTCCTCCTC	(CT)_10_	54	4	130–160	0.726	0.354	0.773
Mean				5		0.683	0.495	0.729

*Ta* annealing temperature, *PIC* polymorphism information content, *bp* base pair, *He* expected heterozygosity, *Ho* observed heterozygosity

### Data analysis

Only clear and unambiguous bands were included in the scoring for creating binary data. SSR profiles were manually checked for scoring the presence or absence of each band. One indicated the presence of band while 0 indicated the absence of band. The polymorphism information content (PIC) value was calculated according to Botstein et al. 1980 [28] for each primer and implemented in program CERVUS version 3.0 [29]. Distance-based cluster analysis was done by generating a dendrogram on the basis of unweighted pair group method of arithmetic mean (UPGMA) using Jaccard’s similarity coefficient with the help of DARwin [30]. The assessment of genetic structure at population level as well as detection of genetic stocks contributing to this germplasm collection was done using Bayesian model-based clustering method implemented in the software structure, version: 2.3.3 [31, 32]. Ancestry model with admixture and correlated allele frequency model was set to get the estimates of posterior probability of data. Ten independent runs were given setting the value of *K* from 1 to 10 with 3 iterations for each value of *K*. Length of burn-in period was set at 100,000 and number of Markov Chain Monte Carlo (MCMC) repeats after burn-in were set at 100,000. Evanno’s method [33]-based program Structure Harvester developed by Earl and Vonholdt [34] was utilized to find the value of estimated Ln probability of data-LnP(K) and to get the best fit value of *K* for the data. Structure was run for all genotypes of the studied species of Asparagus collectively. The values of Fst were also inferred using Structure software.

## Results

### SSR diversity and structure

**Fig. 1:**
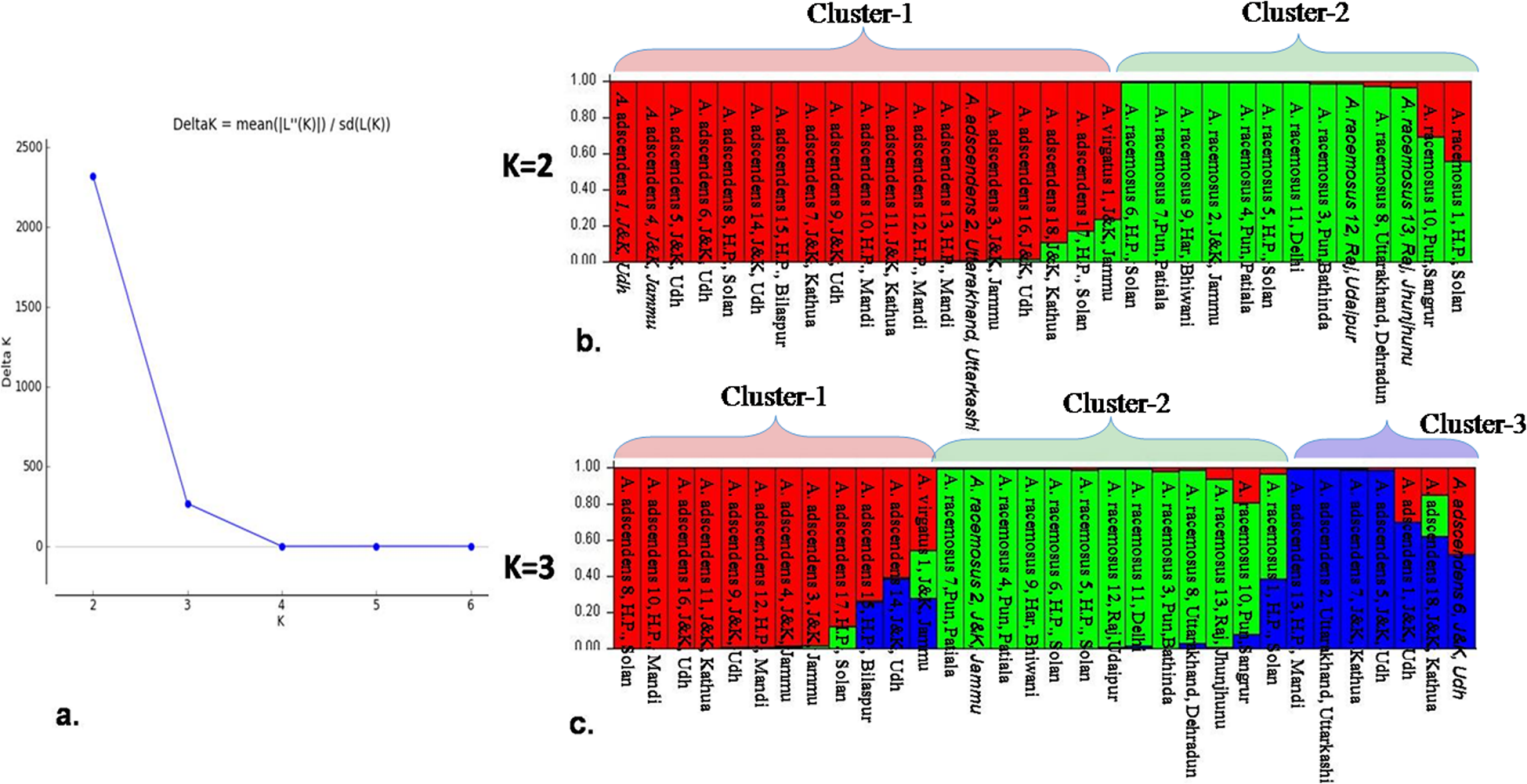
Population genetic structure of *Asparagus* species. **a** Graph showing the best value of *K* at 2. **b** Bar plot of structure at *K* = 2 indicating less admixture between analyzed accessions. **c** Bar plot at *K* = 3 showing two clusters of *A. adscendens* and higher admixture

Twenty-four SSR primers utilized in this study amplified unambiguous and reliable alleles. In total, 24 SSR primers amplified 122 alleles with an average of 5.08 alleles per primer. Size range of alleles varied from 90 bp to 680 bp. Minimum 3 alleles were amplified by three primer pairs, namely, AGA1, SSR-43, and SSR-63. The All SSRs produced reliable and unambiguous alleles. PIC value ranged from 0.389 in primer PN-3 t to 0.790 in SSR 22 primer with an average of 0.683 (Table 2). Similarly, the highest observed heterozygosity (Ho) and expected heterozygosity (He) values of 0.938 and 0.824 were observed in PN-13 and TC1, respectively. Significant Fst values of 0.601 and 0.399 were obtained in cluster 1 and cluster 2 at *K* = 2 and, Fst values of 0.311, 0.399, and 0.230 were observed for the three clusters at *K* = 3. Jaccards similarity matrix of two species (*A. adscendens* and *A. racemosus*) showed maximum dissimilarity value of 0.9744 between *A.adscendens*-13 from Mandi, Himachal Pradesh, and *A.racemosus-*13 from Jhunjhunu, Rajasthan, and minimum of 0.1905 between *A.racemosus*-2 from Jammu and *A.racemosus-*4 form Patiala, Punjab. Structure analysis of these two species showed two populations, and the log likelihood reached a clear maximum value at *K* = 2 (Fig. 1a). The two genetic stocks were revealed by structure analysis and very less admixture was observed (Fig. 1b). However, bar plot of structure analysis at *K* = 3 is also shown in Fig. 1c. At *K* = 3, 19 accessions of *A. adscendens* departed into two groups while 13 accessions of *A. racemosus* remained in one cluster.

### Cluster analysis

**Fig. 2:**
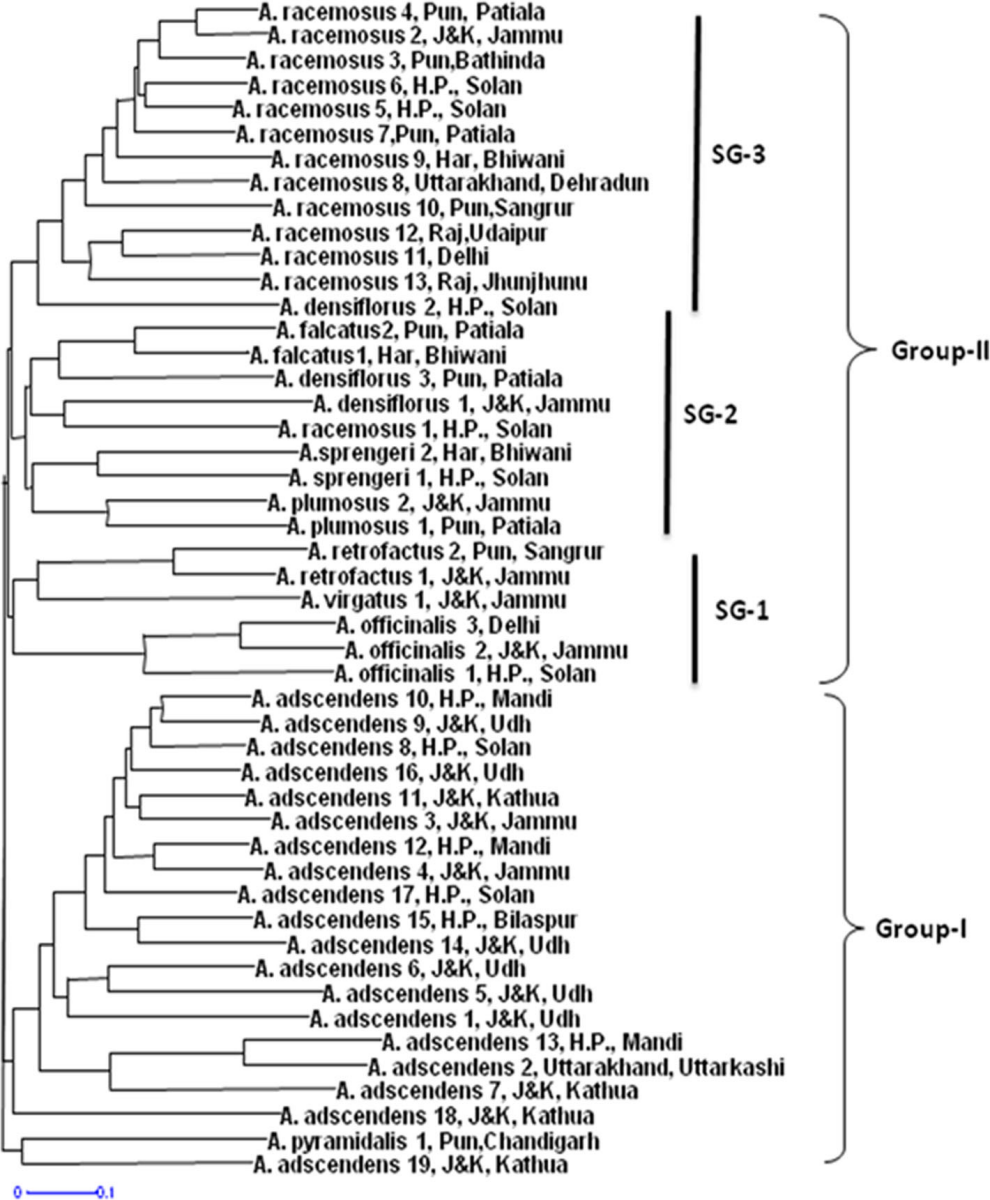
Dendrogram showing relationships among 10 species of *Asparagus*. Group I represents the two species *A. adscendens* and *A. pyramidalis* while group II represents other eight species in three subgroups

In present research work, 122 SSR alleles were used to establish the interrelationships between different species of *Asparagus*. Dendrogram showed two major groups. Group I consisted of 19 accessions of *A. adscendens* and 1 accession of *A. pyramidalis*. Group II consisted of accessions of *A. racemosus* and other seven species, namely, *A. virgatus*, *A. retrofractus*, *A. densiflorus*, *A. officinalis*, *A. plumosus*, *A. sprengeri*, and *A. falcatus*. Further, group II was divided into three subgroups, i.e., SG-I, SG-2, and SG-3.SG-I included six accession in total and three of these were belonging to *A. officinalis*: two of *A. retrofractus* and one of A. virgatus. SG-2 represented nine accessions of five different species, i.e., *A. densiflorus*, *A. plumosus*, *A. racemosus*, *A. sprengeri*, and *A. falcatus*. SG-3 contained 13 accessions belonging to 2 species, i.e., 12 accessions of *A. racemosus* and a single accession of *A. densiflorus*. It was observed that grouping was as per taxonomic ranks rather than the geographic distribution and majority of accessions grouped according to their species boundaries (Fig. 2).

## Discussion

### SSR diversity and structure

Genetic diversity and population structure of *Asparagus* germplasm from India were needed for its improvement in the future. SSR diversity gives the estimates of DNA polymorphism of the analyzed germplasm in the forms of alleles and thereafter the diverse alleles can be used in future for improving the germplasm for various purposes. In this study, primer pair SSR-15 amplified 8 alleles which were double to the alleles amplified by this primer in a study in which it was developed [35]. All other diversity indices, such as average PIC (0.683), Ho (0.495), and He (0.729) indicated high genetic diversity in asparagus germplasm, and hence there are scopes for different improvement works *Asparagus*. Fst values of different populations showed significant structuring of studied germplasm. Structure analysis showed two genetic stocks for the analyzed germplasm and in agreement to the results reported by Lee et al. [36] in *A. cochinchinensis*. The two genetic stocks and very less admixture revealed by structure analysis strongly supported the species level demarcation of accessions. A strong genetic structure was observed in two species, namely, *A. adscendens* and *A. racemosus*. The genetic structuring indicated that *A. racemosus* seem to be more conserved than *A. adscendens*. The conserved nature of *A. racemosus* was also in agreement to earlier study in which low diversity was reported in this species [37]. This genetic structuring showed that despite from distant locations, all accessions remained in the species boundaries. It also indicated the robustness of SSR primers used to analyze the genetic diversity. More such studies with large and distant sampling are required to understand the genetic structure of different *Asparagus* species.

### Cluster analysis

An improved and more reliable method of establishing relationships between individuals is their clustering based on DNA polymorphisms as compared to phenotypic traits. As DNA is merely affected by environmental factors, the relationships revealed by DNA markers can give clear picture of synteny, conserved nature, and differences between analyzed individual of population or different species. A large number of this type of studies based on different DNA markers has been reported previously in various crops [27, 38, 39]. Dendrogram of *A. adscendens* and *A. racemosus* showed two major groups and agreed the results of Altintas et al., Castro et al., and Vijay et al. [36, 40, 41] who also obtained two clades with different *Asparagus* species. Further examination of these groups revealed that group I was showing subgrouping in two subgroups while no such subgroups were observed in group II. It was observed that the sub-clustering was not according to geographical regions but the result of some genetic differentiation due to some other reasons such as retaining of those SSR loci in different populations, which were present in ancestral populations and undergone minor differentiation during evolution. It also indicates that although accessions were distantly located but they did not show adaptive alleles in them. The subgrouping of *A. adscendens* showed highly diverse nature of this species at genetic level. This subgrouping into two clusters was also shown by structure analysis. The clustering pattern of *A. racemosus* clearly indicated that it was genetically most distinct and conserved species. It strongly supports the taxonomic classification of this species based on morphological characters.

In the clustering pattern of the ten species, *A. adscendens* and *A. pyramidalis* showed that both these species are closely related as compared to other eight species included in the study. On the other hand, *A. racemosus* clustered with other seven species showing its close relations to these seven species (*A. officinalis*, *A. plumosus*, *A. sprengeri*, *A. virgatus*, *A. densiflorus*, *A. falcatus*, and *A. retrofractus*). This dendrogram showed *A. adscendens* as distantly related to all *Asparagus* species except *A. pyramidalis*. These distant relations of *A. adscendens* with other species such as A. *racemosus*, *A. densiflorus*, and *A. plumosus* are in contradiction to an earlier study by Idrees et al. 2018 [42]. Chen et al. [43] suggested eight groups based on morphological traits of *A. officinalis* while two groups based on ISSR markers which are in agreement to results of the present study. However, few contradictions in clustering and positioning of some species remain the issue. Therefore, more investigations with improved sampling techniques and different combination of markers are required to establish concrete relationships and phylogeny. The present study is the first comprehensive study which included ten species of *Asparagus* for assessment of genetic diversity and interrelationships. Therefore, the information generated in this study can be useful for other researchers working in the management, breeding, and genetic improvement of different *Asparagus* species throughout the world.

### Improvement strategies and future directions

Various *Asparagus* species are largely cultivated edible purposes in different countries of the world. The sprouts of *Asparagus* species are used as vegetables in food industry. However, in

India, different species of *Asparagus* are mainly grown or exploited for medicinal uses in pharmaceutical industries. Only in few regions like southern states and Uttarakhand the wild stocks of *A. racemosus* are utilized as vegetable [44]. In both the food industry and pharmaceutical industry, the demand of *Asparagus* produce is high due to presence of different secondary metabolites which includes rutin, ascorbic acid, tocopherol, ferulic acid, and glutathione [45]. However, the natural stocks of these species cannot fulfill this demand. Therefore, there is an urgent need of the development of efficient cultivation methods in these species. Besides, identification of the elite germplasm is much more important for mass propagation and genetic improvement of *Asparagus*. Hence, molecular characterization using DNA markers is significant step which will be useful for future improvement strategies through molecular breeding and selection of robust germplasm stocks for large-scale production to meet food and pharmaceutical industry requirements. Our results can be useful in breeding programs of asparagus because they can help in identification of diverse genotypes with which we can precede for breeding methods. However, other types of associated data will also be beneficial to be taken into consideration such as biochemical and phenotypic data of genotypes.

The present work is, therefore, applicable to initiate such improvement programs in *Asparagus* species in the country which can result in development of new cultivars and improved varieties as per requirement of industries and consumers.

## Conclusion

In the present work, 48 accessions belonging to 10 different species of *Asparagus* were characterized using 24 SSR markers. Of these, eight SSR markers were newly developed in this study. All SSR markers were polymorphic and revealed high genetic diversity in studied accessions of different *Asparagus* species. Results showed that there are 2 genetic stocks contributing to the genetic makeup of all the 10 species. *A. adscendens* and *A. pyramidalis* were found closely related. The findings of this research work can be useful in identification of promising genotypes for large-scale production and for initiating new improvement programs in *Asparagus* species to meet the industries demands.

## Supplementary information

**Additional file 1: Supplementary Table 1.**. Accession numbers of the specimens deposited in Herbaria of Punjabi University, Patiala (PUN). **Supplementary Table 2.** Details of each Primer showing null alleles in different accessions

## Data Availability

Not applicable

## Abbreviations

SSR: Simple sequence repeat
PIC: Polymorphism information content
CTAB: Cetyle trimethyle ammonium bromide
Ta: Annealing temperature
Bp: Base pairs
PCR: Polymerase chain reaction
dNTPs: Deoxy nucleoside triphosphate
