# Supplementary material for: Analysis of genetic diversity and population structure in Asparagus species using SSR markers
Source: J Genet Eng Biotechnol. 2020 Sep 14;18:50. doi: 10.1186/s43141-020-00065-3 (PMC7490301; doi:10.1186/s43141-020-00065-3)
Supplement: Supplementary file 1 — Additional file 1: Supplementary Table 1.. Accession numbers of the specimens deposited in Herbaria of Punjabi University, Patiala (PUN). Supplementary Table 2. Details of each Primer showing null alleles in different accessions [file 43141_2020_65_MOESM1_ESM.docx]

**Supplementary Table1:**

Accession numbers of the specimens deposited in Herbaria of Punjabi University, Patiala (PUN).

| **Species** | **Locality** | **Accession no.** | **Altitude (m)** |
| --- | --- | --- | --- |
| *A. racemosus* Willd. | Udaipur, Rajasthan | 62456 | 600 |
| *A. adscendens* Roxb. | Udhampur, J&K | 62457 | 755 |
| *A.* *officinalis* L. | Solan, H.P. | 62458 | 1502 |
| *A. sprengeri* Regel | Bhiwani, Haryana | 62459 | 225 |
| *A. retrofractus* L. | Jammu, J&K | 62460 | 305 |
| *A. densiflorus* (Kunth) Jessop | Patiala, Punjab | 62461 | 244 |
| *A. falcatus* L.  *A. plumosus* Baker  *A. pyramidalis* Baker  *A. virgatus* Baker | Patiala, Punjab  Chandigarh  Jammu, J&K  Jammu, J&K | 62462 | 244 |

**Supplementary Table 2:**

Details of each Primer showing null alleles in different accessions.

| **S. No.** | **Primer Name** | **Accessions in which null allele detected (S. No. of accession is as per Table 1)** |
| --- | --- | --- |
|  | SSR-13 | 1,5,6,7,13 |
|  | SSR-15 | 11 |
|  | SSR-22 | 2,7, 18,39,43 |
|  | SSR-37 | 14,23,35 |
|  | SSR-40 | 7, 18,38,42,45,46 |
|  | SSR-43 | 1,18,33,34,35,36,37,38,39,40, 48 |
|  | SSR-56 | 23,32,37,40 |
|  | SSR-63 | 1, 29,39,32,47 |
|  | SSR-69 | 7, 24,38,40,41,42, 43, 47 |
|  | SSR-77 | 2, ,8,13,15,27,31,44 |
|  | SSR-83 | 4,11,13,16,33 |
|  | PN3 | 3,14,16,39 |
|  | PN4 | 1,2,5,6,13,35,36,38,46 |
|  | PN9 | 14,15,35 |
|  | PN10 | 2,7 |
|  | PN12 | 1, 25,27 |
|  | PN13 | 2,13 |
|  | PN14 | 30,43,45,46 |
|  | PN15 | 14,15,16,17,33,37,47,48 |
|  | AGA1 | 2 |
|  | AG5 | 23,40,41,44,46,48 |
|  | TC1 | 2,5,7,9,28,32 |
|  | TC3 | 12,35 |
|  | TC8 | 19,20,28,29,33,34,38,39,43,46,47 |
